# Efficacy of bupropion and varenicline genetic markers in choosing pharmacological treatment for smoking cessation, and implications for combining drugs: A randomized controlled trial – GENTSMOKING

**DOI:** 10.18332/tid/186072

**Published:** 2024-04-16

**Authors:** Patricia V. Gaya, Juliana R. Santos, Paulo R. X. Tomaz, Tania M. O. Abe, Miguel Nassif, Larissa G. Galas, Bianca B. Bellini, Iana R. Moraes, Paulo C. Lima Santos, Paulo C. R. P. Correa, Jaqueline R. Scholz

**Affiliations:** 1 Programa de Tratamento do Tabagismo do Servico de Prevencao e Reabilitacao, Instituto do Coracao, Hospital das Clinicas HCFMUSP, Faculdade de Medicina, Universidade de Sao Paulo, Sao Paulo, Brazil; 2 Laboratorio de Genetica e Biologia Molecular, Instituto do Coracao, Hospital da Clinicas da Faculdade de Medicina da Universidade de Sao Paulo, Sao Paulo, Brazil; 3 Departamento de Farmacologia, Escola Paulista de Medicina, Universidade Federal de Sao Paulo, Sao Paulo, Brazil; 4 Universidade Federal de Ouro Preto, Minas Gerais, Brazil

**Keywords:** precision medicine, varenicline, bupropion, smoking cessation, pharmacogenetics

## Abstract

**INTRODUCTION:**

Smoking cessation is the best strategy for reducing tobacco-related morbimortality. The goal of this randomized controlled trial was to test whether using the genetically favorable markers to choose a smoking cessation drug treatment (precision medicine) was superior to using the most effective drug (varenicline) in terms of abstinence rates. Additionally, combination therapy was tested when monotherapy failed.

**METHODS:**

This partially blind, single-center study randomized (1:1) 361 participants into two major groups. In the genetic group (n=184), CYP2B6 rs2279343 (genotype AA) participants started treatment with bupropion, and CHRNA4 rs1044396 (genotype CT or TT) participants started treatment with varenicline; when genetic favorable to both, participants started treatment with bupropion, and when favorable to neither, on both drugs. In the control group (n=177), participants started treatment with varenicline, regardless of genetic markers. Drug treatment lasted 12 weeks. Efficacy endpoints were abstinence rates at Weeks 4, and Weeks 8–12, biochemically validated by carbon monoxide in exhaled air. Participants who did not achieve complete abstinence at Week 4, regardless of group, were given the choice to receive combination therapy.

**RESULTS:**

Abstinence rates were 42.9% (95% CI: 36–64) in the control group versus 30.4% (95% CI: 23–37) in the genetic group at Week 4 (p=0.01); and 74% (95% CI: 67–80) versus 52% (95% CI: 49–64) at Week 12 (p<0.001), respectively. The strategy of combining drugs after Week 4 increased abstinence rates in both groups and the significant difference between genetic and control groups was maintained.

**CONCLUSIONS:**

Results show that using these selected genetic markers was inferior to starting treatment with varenicline (control group), which is currently the most effective smoking cessation drug; moreover, the addition of bupropion in cases of varenicline monotherapy failure improves the efficacy rate until the end of treatment.

**CLINICAL TRIAL IDENTIFIER:**

NCT03362099

## INTRODUCTION

Smoking is the leading cause of preventable death globally^1,2^ and of cancer death in the US^3^. It has been associated with increased rates of cardiovascular and respiratory diseases; current smokers have shown increased cardiovascular risk compared to former smokers^4,5^, including those with a long and heavy history of tobacco consumption^4^. Smoking cessation is one of the most essential strategies to reduce general smoking-related morbimortality^6^.

Pharmacological smoking cessation therapies have been shown to double the chances of quitting successfully when administered in adjunction to brief physician counselling^7^. Unfortunately, a limited number of drugs are available for use in such therapies. First-line oral drugs approved by the US Food and Drug Administration are bupropion, a norepinephrine/dopamine-reuptake inhibitor, and varenicline, an alpha4beta2 nicotinic receptor partial agonist^7^. Previous studies indicated that bupropion-treated smokers showed higher abstinence rates compared to placebo; in a systematic review, Hajizadeh et al.^8^ stated that there is high-certainty evidence that bupropion increases long-term smoking cessation rates. Varenicline, on the other hand, is more effective than bupropion and other smoking cessation treatments, such as nicotine patches^9^.

Combination therapies should be considered for improving treatment efficacy, despite the possibility of increased adverse event rates and high treatment costs^10,11^. As patients respond differently to drug treatments, reaching an ideal individual response for each patient is challenging; in this scenario, precision medicine is a helpful tool for smoking cessation treatment design^12^. In the last decade, there has been significant improvement in genetic studies discussing patient responsiveness to drugs, including treatment efficacy and safety profile^13,14^, and the differences in individual responses have become the subject of specific investigation in pharmacogenetic studies^13^.

Two previous investigations on genetic markers related to better treatment outcomes were conducted with varenicline^15^ and bupropion^16^ considering a smoking cessation database [Programa de Assistência ao Fumante (Smoker Assistance Program)]^17^ with over nine hundred smokers treated between 2007 and 2013. Santos et al.^15^ showed a positive relation between *CHRNA4* rs1044396 polymorphism and varenicline treatment success. *CHRNA4* is an important gene for smoking cessation pharmacogenetic studies as it encodes the alpha4 subunits of nicotinic acetylcholine receptors, which are an important target for varenicline^18^. Participants with mutant CT or TT genotypes for *CHRNA4* rs1044396 had significantly higher success rates with varenicline than participants with wild-type CC genotype. Tomaz et al.^16^ described an association between *CYP2B6* rs2279343 polymorphism, involved in the coding of drug metabolizing enzymes, and bupropion treatment efficacy. Cytochrome P450 CYP2B6 is the main isoenzyme involved in bupropion metabolism^19^, and this study indicated that participants with mutant AA genotype had significantly higher success rates compared to those with AG or GG genotypes.

The prospective, randomized controlled trial GENTSMOKING aimed to evaluate whether considering varenicline and bupropion genetically favorable markers in the choice of an optimized smoking cessation drug treatment (precision medicine) was superior to treatment with the current most effective drug (varenicline) in terms of abstinence rates. Moreover, the study evaluated the effects of combination therapy (varenicline + bupropion) on the success rate of smoking cessation after monotherapy failure. Primary endpoints were biochemically validated smoking cessation rates at Week 4 and continuous abstinence rates at Weeks 8–12.

## METHODS

### Study design

This was a single-center, partially blind, randomized controlled study conducted between January 2017 and April 2022. This study was approved by the Research Ethics Committee of the Heart Institute in São Paulo, Brazil, and was carried out in accordance with the ethical principles of the Declaration of Helsinki and applicable international, national and/or institutional guidelines. All study participants provided written informed consent. 2010 CONSORT guidelines^20^ were followed for reporting of this study.

### Study population

This study enrolled current smokers (at least five cigarettes/day in the previous year) aged 18–75 years seeking out smoking cessation treatments at the local site. Individuals with clinically stable diseases, e.g. depression or anxiety disorders stable for over three months, were included. Subjects were excluded if any of the following criteria were met: alcohol or illicit drug consumption; risk of pregnancy; significant liver, renal, or gastrointestinal diseases; unstable cardiovascular or psychiatric disorders; seizures or risk of convulsion; head trauma or brain tumor; previous allergic reactions to bupropion or varenicline; and unwilling to follow study procedures and schedules.

### Genotyping

Eight mL of peripheral whole blood samples were collected into anticoagulant-coated (Ethylenediaminetetraacetic Acid Tripotassium Salt) BD Vacutainer^®^ (0.15mg/mL, Becton Dickinson, NJ, USA) tubes. Genomic DNA extraction from leukocytes was carried out using QIAamp^®^ DNA Blood Mini Kit (QIAGEN, SP, Brazil).

The *CHRNA4* gene rs1044396 polymorphism was genotyped by real-time quantitative polymerase chain reaction (PCR) using TaqMan^®^ (C__25765540_10) (Applied Biosystems, CA, USA) and Rotor-Gene^®^ Q (QIAGEN, SP, Brazil). The *CYP2B6* gene rs2279343 polymorphism was genotyped according to Lang et al.^21^: traditional PCR followed by enzymatic restriction using StyI-HF^®^ (New England BioLabs, MA, USA). Genotyping results were obtained using agarose gel electrophoresis.

Hardy-Weinberg equilibrium analyses were conducted using the Court-Lab-HW^22^ calculator to evaluate the genotypic distribution of each polymorphism. A p>0.05 is considered consistent with the Hardy-Weinberg equilibrium.

### Study procedures and interventions

The study began with a screening visit, in which participants signed a written informed consent form agreeing to participate. Blood samples were collected from all participants for polymorphism genotyping. Clinical and smoking histories were recorded, and nicotine dependence was measured using the Fagerström test for nicotine dependence^23^ and scored according to Issa^24^. A total of seven visits were conducted on-site (screening, baseline, weeks 2, 4, 6, 8, and 12) for evaluation of concomitant medications; carbon monoxide (CO) concentration (ppm) in exhaled air using Smokerlyzer^®^ (Bedfont Scientific Ltd., Maidstone, UK); nicotine inventory questionnaire; weight; blood pressure and heart rate using a semi-automatic oscillometric digital device); and adverse events.

Randomization 1:1 of screened participants into two arms (control group and genetic group) was done by a genetic team using a computer-generated sequence. Even though all participants were submitted to genetic testing at screening, only in the genetic group were polymorphisms used to define smoking cessation drug prescription (varenicline 2 mg, bupropion 300 mg, or combination therapy with varenicline 2 mg + bupropion 150 mg; commercial drug products). The genetic team was responsible for indicating to the medical staff which drug would be prescribed to each participant at the beginning of treatment (baseline visit), around 2 to 4 weeks after screening. The control group was set to receive varenicline, while the genetic group would start treatment with varenicline or bupropion (for *CHRNA4* rs1044396 (genotype CT or TT) or *CYP2B6* rs2279343 (genotype AA), respectively); when both genetic markers were present, participants were started on bupropion, and when neither of genetic markers were found, participants were started on both drugs simultaneously. As such, participants and medical staff were blind only to varenicline administration. Varenicline was supplied as 1 mg tablets for twice-a-day administration (2 mg/day); bupropion was supplied as 150 mg tablets for twice-a-day administration (300 mg/day); both medications were subjected to dose escalation.

Drug treatment lasted a total of 12 weeks. Participants were encouraged to reduce cigarette consumption during the first week and to try to quit smoking after the second week. ‘Cue restricted smoking’^25^ was suggested in cases of inability to stopping smoking due to nicotine cravings. This technique is based on lessening stimuli for smoking: smokers are supposed to smoke alone while standing and facing a wall, without any distractions (e.g. drinks, food, cell phones). At Week 4, all participants from monotherapy regimens who had failed to stop smoking by then, were started on combination therapy to increase treatment efficacy. Thus, participants taking varenicline had bupropion 150 mg added to their daily regimen, while participants taking bupropion had their daily dose reduced to 150 mg and varenicline 2 mg added to their daily regimen.

### Study endpoints and analysis

The primary efficacy endpoints of the study were the abstinence rate at Week 4 and the continuous abstinence rate at Weeks 8–12, respectively, both biochemically validated by CO in exhaled air. The cut-off for smoking cessation was a CO concentration of ≤3 ppm ^26^ for the genetic group versus the control group.

Study secondary efficacy endpoints (subgroups analyses) were abstinence rates evaluated according to drug cohorts (drug used to start treatment, varenicline 2 mg/day or bupropion 300 mg/day) and therapy subgroups [monotherapy or combination therapy starting at baseline or later (varenicline 2 mg/day + bupropion 150 mg/day)] regardless of randomization group (genetic group or control group).

The most effective strategy for combining smoking cessation drugs, in case of monotherapy failure, was studied using the cohort of participants starting treatment with both drugs at baseline as a reference, considering treatment discontinuation according to the medication used. Another goal of the study was to identify predictive variables for treatment efficacy. Regarding safety objectives, clinically observed or self-reported adverse events and treatment discontinuations related to side effects were recorded. The intent-to-treat principle was applied to result analyses.

### Statistical analysis

Sample size calculation indicated that at least 160 participants were required in each group for 80% power at an alpha level of 5% for Type I error to detect a 15% statistical difference between study groups (control group and genetic group, proportions p_1_=0.30 and p_2_=0.45, respectively). To account for a possible 5% sample loss, 361 participants were required for randomization. A power/sample size calculator was used^27^.

Efficacy and safety analyses were conducted on randomized participants who received at least one dose of study treatment (intent-to-treat analysis). Data are expressed descriptively. For categorical variables, absolute and relative frequencies are presented, and summary measures (mean and standard deviation) for numerical variables.

The presence of associations between two categorical variables was tested using the chi-squared test, or Fisher’s exact test in cases of small samples. Mean comparisons between groups were performed using Student’s t-test. Normality in data distribution was verified using the Kolmogorov-Smirnov test. In case of violation of this assumption, a non-parametric Mann-Whitney test was used.

Univariable and multivariable logistic regression models are used to assess treatment efficacy, considering the control group as a reference. The following variables were included in this model to verify possible effects on results: bupropion-favorable polymorphism, varenicline-favorable polymorphism, sex, age, number of cigarettes consumed at baseline, baseline carbon monoxide concentration, baseline heart rate, depression, anxiety, antidepressants/anxiolytics use, Fagerström test, Issa score, concomitant medications, chronic obstructive pulmonary disease, diabetes mellitus, systemic arterial hypertension, and other diseases.

In subgroup analysis, the effect of drugs on overall success was assessed via a logistic regression model adjusted for polymorphism (bupropion-favorable polymorphism, varenicline-favorable polymorphism), sex, age, baseline carbon monoxide concentration, baseline heart rate, depression, anxiety, antidepressants/anxiolytics use, Fagerström test, Issa score, concomitant medications, chronic obstructive pulmonary disease, diabetes mellitus, systemic arterial hypertension, other diseases, and adverse events, taking the population on bupropion monotherapy as reference. *Ad hoc* tests (Wald test) performed based on the final multivariable model were corrected using the Bonferroni method to maintain a global significance level.

The delta of consumption at Week 4 of treatment was calculated from: [(cigarettes per day at baseline - cigarettes per day at Week 4)/cigarettes per day at baseline] × 100%. This parameter was included in the model as a mediating variable, as it was influenced by the drug used in smoking cessation treatment. To this end, a normal distribution was assumed for this variable. The model was estimated via generalized structural equations (General Structural Equation Modeling).

For all statistical analyses, a significance level of 5% was considered (2-tailed testing). Results from logistic regression analyses were expressed using odds ratio (OR) and 95% confidence interval (95% CI). Statistical packages SPSS 20.0 and STATA 17 were used.

## RESULTS

**Figure 1:**
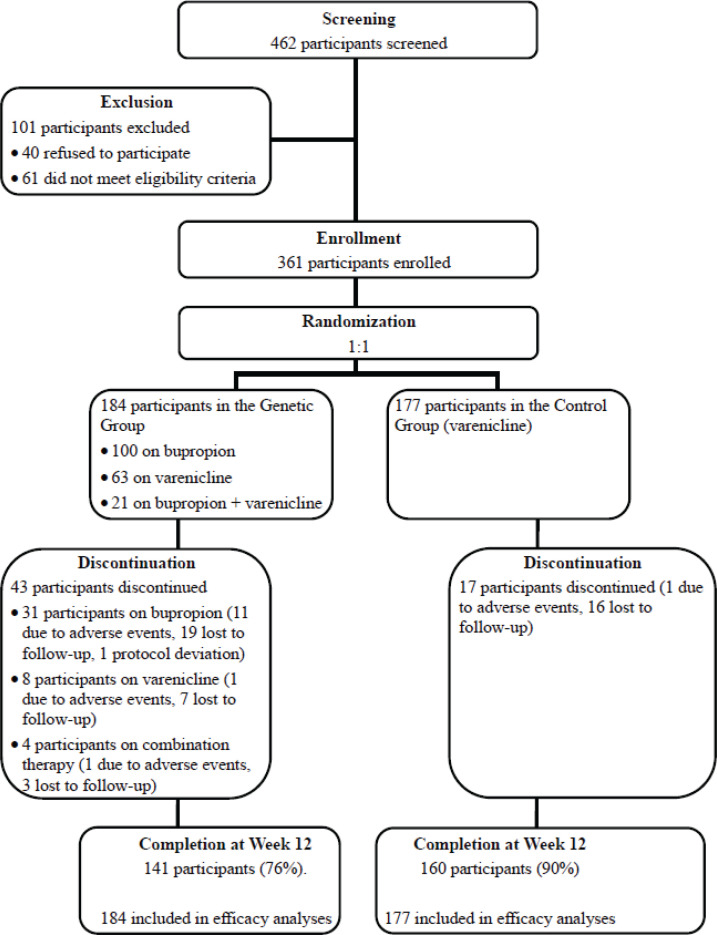
Study flow diagram including screening, enrollment, randomization, discontinuation, and completion

A total of 462 participants were screened, and 361 were randomized: 177 to the control group and 184 to the genetic group. The study flowchart is shown in Figure 1. Demographics, clinical characteristics, previous psychiatric comorbidities, cigarette consumption, and nicotine dependence were comparable between groups (Table 1). For CHRNA4 gene rs1044396 polymorphism, variant allele (T) frequency was 45.8%, and genotype distribution was 27.7% (n=100) for CC, 52.9% (n=191) for CT, and 19.4% (n=70) for TT. For CYP2B6 gene rs2279343 polymorphism, variant allele (C) frequency was 27.3%, and genotype distribution was 52.4% (n=189) for AA, 40.7% (n=147) for AG, and 6.9% (n=25) for GG. Genotypic distribution for CHRNA4 rs1044396 and CYP2B6 rs2279343 was in accordance with the Hardy-Weinberg equilibrium^22^ (χ^2^=1.55 and p=0.21, and χ^2^=0.25 and p=0.62, respectively).

**Table 1:** Demographic and clinical characteristics, smoking history, and nicotine consumption, in the control and genetic groups

*Characteristics*	*Control group (N=177) n (%)*	*Genetic group (N=184) n (%)*	*p*
Female	111 (62.7)	109 (59.2)	0.49
Age (years), mean ± SD	51.9 ± 12.0	51.1 ± 11.4	0.34
Caucasian	151 (85.3)	161 (87.5)	0.78
Body mass index (kg/m^2^), mean ± SD	26.95 ± 4.73	26.59 ± 4.31	0.44
Diabetes	23 (13.0)	13 (7.1)	0.06
Hypertension	58 (32.7)	55 (29.8)	0.20
Coronary disease	9 (5.1)	15 (8.2)	0.24
Depression	24 (13.6)	23 (12.5)	0.76
Anxiety	55 (31.1)	49 (26.6)	0.35
Dyslipidemia	54 (30.5)	47 (25.5)	0.29
Chronic obstructive pulmonary disease	17 (9.6)	21 (11.4)	0.57
Other diseases	56 (31.6)	54 (29.3)	0.63
Serotonin reuptake inhibitors	79 (44.6)	76 (41.3)	0.52
Other drugs, mean ± SD	2.1 ± 1.8	1.8 ± 1.7	0.12
Number of cigarettes/day, mean ± SD	20.6 ± 9.0	18.9 ± 7.9	0.06
Carbon monoxide (ppm), mean ± SD	12.6 ± 5.7	12.4 ± 5.6	0.70
Fagerström test, mean ± SD	6.8 ± 2.13	6.71 ± 1.8	0.50
Issa score, mean ± SD	3.2 ± 0.6	3.1 ± 0.6	0.50
Bupropion-favorable genetic marker	89 (50.3)	100 (54.3)	0.43
Varenicline-favorable genetic marker	129 (72.9)	132 (71.7)	0.80

### Efficacy

The proportion of participants who quit smoking at Week 4 was significantly greater in the control group compared to the genetic group (42.9%, 76/177; 95% CI: 35–50, and 30.4%, 56/184; 95% CI: 24–37, p=0.014, respectively). Continuous abstinence rates at Weeks 8–12 in the control group were significantly higher compared to the genetic group (74%, 132/177; 95% CI: 62–76, and 57%, 105/184; 95% CI: 49–64, p=0.001, respectively) (Table 2). Logistic regression adjusted for demographics, clinical characteristics, previous psychiatric disorders, and nicotine dependence showed that the chance of treatment success in the genetic group was 52% lower at Week 4 (0.48; 95% CI: 0.30–0.78, p=0.003) and 60% lower at Weeks 8–12 (0.40; 95% CI: 0.25–0.65, p<0.001) when compared to the control group.

**Table 2:** Logistic regression analysis using univariable and adjusted models for comparison of efficacy, in the control and genetic groups at Week 4 and Weeks 8–12

			*Univariable model*		*Adjusted model*	
	*n/N (%)*	*95% CI*	*OR (95% CI)*	*p*	*AOR (95% CI)*	*p*
**Abstinence rate at Week 4**						
Genetic group	56/184 (30.4)	23.9–37.6	0.58 (0.38–0.90)	0.014	0.48 (0.30–0.78)	0.003
Control group ®	76/177 (42.9)	35.5–50.6	1		1	
**Abstinence rate at Weeks 8–12**						
Genetic group	105/184 (57.1)	49.6–64.3	0.45 (0.29–0.71)	0.001	0.40 (0.25–0.65)	<0.001
Control group ®	132/177 (74.6)	67.5–80.8	1		1	

AOR: adjusted odds ratio; model adjusted for demographics, clinical and psychiatric comorbidities, concomitants medication, cigarette consumption, nicotine dependence scores, bupropion-favorable genetic marker, and varenicline-favorable genetic marker. ® Reference categories.

### Subgroups analysis

Given that the primary hypothesis of this study was null, subgroup analyses were conducted considering drugs received, polymorphism types, and treatment outcomes. The presence of drug-favorable polymorphisms did not improve the efficacy of either varenicline or bupropion (Supplementary file Table 1). There were significant differences in abstinence rates for participants who started treatment with bupropion compared to those who started with varenicline and varenicline + bupropion. There was no meaningful difference in abstinence rates at Week 4 between participants treated with varenicline monotherapy, regardless of genetic polymorphisms, and those who received varenicline + bupropion (Supplementary file Table 1).

For varenicline-treated participants: 240 participants started treatment (177 in the control group plus 63 in the genetic group), 103 stopped smoking by Week 4 (5 relapsed before the end of the study), and 25 participants discontinued treatment. Seventy-nine participants agreed to combination therapy (addition of bupropion), 49 of whom achieved the status of smoking cessation; 33 participants did not agree to combination therapy and were maintained on varenicline alone, and 32 achieved the status of smoking cessation. Treatment success rate at Weeks 8–12 in this subgroup was 74% (179/240).

For bupropion-treated participants: 100 participants started treatment in the genetic group, 19 stopped smoking by Week 4, and 31 participants discontinued treatment. Thirty-nine agreed to combination therapy (addition of varenicline), 25 achieved smoking cessation; 11 participants did not agree to combination therapy and were maintained on bupropion alone, 1 stopped smoking, and 2 relapsed by Week 12. Treatment success rate at Weeks 8–12 in this subgroup was 43% (43/100).

For participants on combination therapy (varenicline plus bupropion) since baseline: 21 participants started treatment, and 10 stopped smoking by Week 4. Four participants discontinued treatment, while 7 remained on treatment despite failing to achieve smoking cessation at Week 4. Five participants were able to stop smoking by Weeks 8–12, of which none relapsed. The treatment success rate at Weeks 8–12 in this subgroup was 71% (15/21).

### Drug combination

Regarding the efficacy of combination therapy, participants starting treatment with varenicline plus bupropion at baseline (n=21) were compared to those starting with varenicline at baseline and then adding bupropion at Week 4 (n=79), as well as to those starting treatment with bupropion and then adding varenicline at Week 4 (n=39). There was no significant difference in abstinence rates between groups (p=0.72). Efficacy rates were 71.4% (15/21; 95% CI: 47.8–88.7) for the reference arm, versus 62.2% (49/79; 95% CI: 50.4–72.7) for bupropion added to varenicline at Week 4 (n=79) and 64.1% (25/39; 95% CI: 46–78.2) for varenicline added to bupropion at Week 4 (n=39) (Supplementary file Table 2).

Although there was no meaningful difference in abstinence rates for participants with distinct drug combination strategies, a significant increase was observed in treatment discontinuation by participants who started treatment with bupropion (n=31/100; 31%) compared to those receiving varenicline at baseline (n=25/240; 10.4%, p<0.001). Bupropion-treated participants were 3.54 times more likely to discontinue treatment than varenicline-treated participants (95% CI: 1.98–6.35, p<0.001). There was no difference in discontinuation treatment rates between participants who started treatment with varenicline at baseline and then added bupropion at Week 4 compared to those receiving varenicline alone (n=4/21; 19%; p=0.296) (Supplementary file Table 3).

### Predicted variables

None of the variables related to demographics, clinical comorbidities, history of psychiatric disorders, smoking history, nicotine dependence, or favorable polymorphism was able to help predict the efficacy of smoking cessation treatments in this study. However, a subgroup of participants stood out; they were on varenicline monotherapy, were incapable of stopping smoking by Week 4, and refused the addition of bupropion to their drug regimen, stating that cigarette consumption had been greatly reduced by Week 4 and that they felt they would be able to stop smoking in the next few days. In that regard, cigarette consumption reduction was evaluated for participants receiving monotherapy who did not stop smoking by Week 4, comparing those who agreed to combination therapy versus those who did not. Table 3 shows delta of consumption values considering the drugs used throughout 12 weeks of treatment. Delta of consumption was higher among participants on varenicline monotherapy and lower among those who required combination therapy or were on bupropion monotherapy. This new variable was tested in the prediction model for all participants’ treatment success by Week 12 (overall success rate) (Table 4).

**Table 3:** Delta (%) of consumption at Week 4, considering the medications that were used throughout the entire 12-week treatment period (N=361)

*Drug*	*Mean ± SD*	*n*
Varenicline	86.5 ± 22.4	161
Bupropion	57.8 ± 36.7	62
Varenicline and bupropion at baseline	76.9 ± 31.3	21
Background varenicline plus bupropion add-on	62.4 ± 19.0	79
Background bupropion plus varenicline add-on	50.3 ± 23.2	38

**Table 4:** Logistic regression analysis for overall success of treatment considering drug used in Model 1 and adjusted for different variables, using an estimate of generalized structural equation system for delta of consumption in Model 2 (N=361)

	*Model 1*		*Model 2*	
	*OR (95% CI)*	*p*	*AOR (95% CI)*	*p*
**Treatment success at Weeks 8–12**				
**Drug** (Ref. bupropion)		<0.001		<0.001
Varenicline	9.49 (4.87–18.49)	<0.001	4.86 (1.81–13.08)	0.002
Varenicline and bupropion	5.66 (1.90–16.83)	0.002	4.26 (1.70–25.94)	0.116
Background varenicline plus bupropion add-on	3.70 (1.83–7.49)	<0.001	6.01 (2.15–16.80)	0.001
Background bupropion plus varenicline add-on	3.88 (1.65–9.10)	0.002	15.89 (4.80–52.56)	<0.001
**Variables included in logistic regression Model 2**				
Delta of consumption at Week 4			1.06 (1.04–1.07)	<0.001
Bupropion-favorable genetic marker			0.71 (0.34–1.49)	0.362
Varenicline-favorable genetic marker			0.74 (0.36–1.54)	0.423
Male (Ref. female)			0.92 (0.48–1.75)	0.799
Age (years)			0.99 (0.96–1.02)	0.619
Baseline CO in exhaled air			0.97 (0.92–1.03)	0.342
Baseline heart rate			0.99 (0.96–1.02)	0.405
Depression			2.10 (0.63–6.93)	0.225
Anxiety			1.14 (0.43–3.02)	0.786
Serotonin reuptake inhibitors			0.93 (0.33–2.59)	0.885
Fagerström test for nicotine dependence			1.04 (0.48–2.26)	0.915
Issa score			0.87 (0.32–2.38)	0.780
Number of additional medications			0.87 (0.68–1.13)	0.302
Chronic obstructive pulmonary disease			0.63 (0.24–1.63)	0.339
Diabetes			0.94 (0.34–2.57)	0.905
Systemic arterial hypertension			1.26 (0.56–2.80)	0.578
Other diseases			1.77 (0.86–3.65)	0.119
On-treatment adverse events			1.08 (0.60–1.96)	0.795

AOR: adjusted odds ratio.

Statistical analyses considered the drug effects on overall success rate using a logistic regression model, adjusted for bupropion-favorable polymorphism, varenicline-favorable polymorphism, gender, age, CO in exhaled air and heart rate at baseline, depression, anxiety, antidepressants/anxiolytics use, Fagerström test, Issa score, concomitant medications, chronic obstructive pulmonary disease, diabetes, hypertension, other diseases, and adverse events, considering bupropion monotherapy as reference. The model included the delta of consumption at Week 4 as a mediating variable, and normal distribution was assumed. Models were estimated by a generalized structural equation modeling system (Table 4). Model 1 had no adjustments, while Model 2 was adjusted for variables. In addition, Model 2 considered an indirect effect of the drug treatment group on the relative risk for delta of consumption at Week 4 (p<0.001) and a direct effect on overall success rate (p<0.001). As such, participants on treatment with varenicline monotherapy or varenicline + bupropion since baseline had a much higher chance of achieving smoking cessation than participants who started treatment with bupropion. These changes increased only for participants who started treatment with bupropion and then added varenicline at Week 4.

In addition to drug type, the delta of consumption was the only other helpful variable to predict the chance of treatment success (p<0.001); an increase of 1 percentage point in cigarette consumption reduction led to a 6% increase in the chance of overall success.

### Drug safety

Of the 361 participants that were randomized into the study, 24 discontinued treatment due to adverse events. Among varenicline-treated participants (n=240), three discontinued treatment due to nausea and one due to vivid dreams. Among bupropion-treated participants at 300 mg/day (n=100), 12 participants discontinued treatment: 5 due to headache, 4 due to insomnia, 2 due to irritability, and one due to tremors. Among participants starting treatment with both drugs (varenicline 2 mg/day + bupropion 150 mg/day) at baseline (n=21), one discontinued due to insomnia. Despite drug discontinuation, 1 participant receiving varenicline and 1 participant receiving bupropion 300 mg/day remained abstinent at Weeks 8–12 (Supplementary file Table 4). The frequency of adverse events was 60% for all drug treatments in the first four weeks. Adverse event rates with varenicline and bupropion agreed with those from previous studies^9^. After Week 4, adverse event frequencies were reduced in participants who kept on their initial drug regimen, and higher values were observed among those who added varenicline or bupropion. New users of bupropion 150 mg discontinued treatment more frequently than new users of varenicline, although less frequently than those on bupropion 300 mg since baseline. Participants who started treatment with both drugs at baseline reported more adverse events; nausea was the most frequent one. However, it did not lead to treatment discontinuation (Supplementary file Table 5).

## DISCUSSION

This study evaluated the role of specific genetic markers in choosing drug treatment for smoking cessation in a randomized, partially blinded design. The rationale for this study is supported by genomic information obtained from a previous observational study at our site. As such, this study is one of the few that evaluated the predictive value of genetic markers using a randomized and prospective design in which participants were eligible for treatment with a selected drug, if they had a favorable genetic variable. Results showed that prescribing smoking cessation drugs based on *CYP2B6* rs2279343 (genotype AA) or *CHRNA4* rs1044396 (genotype CT or TT) pharmacogenomic variables, that are in theory associated with better responses to bupropion or varenicline, was inferior to prescribing the most effective smoking cessation drug currently available, varenicline, in terms of treatment success.

Previous studies of pharmacogenomic variables indicated great heterogenicity in this type of investigation; many have failed to find eligible pharmacogenomic variables^28^, especially those related to bupropion and varenicline, as critically addressed by Chen et al.^29^. The evidence gaps for confirming the quality of genomic variables for smoking cessation are probably related to possible selection bias in genotyping trial participants. This is what is thought to have occurred in previous studies indicating *CYP2B6* rs2279343^16^ and *CHRNA4* rs1044396^15^ as possible markers of better responses to bupropion and varenicline: selection bias within the original dataset of 900 patients submitted to smoking cessation treatment between 2007 and 2013. Of these, only 478 participants underwent blood sampling for genetic analysis. When considering that the frequency of both polymorphisms is high (almost 50% for bupropion (genotype AA) and 70% for varenicline (genotypes CT or TT)), it is possible that participants who were willing to undergo blood sampling for genetic purposes were part of the subgroup most successful at smoking cessation treatment.

The limitation of using genetic markers for smoking cessation has been discussed by Panagiotou et al.^30^. The authors performed a systematic review and meta-analysis including 9017 non-Hispanic black and white smokers with *CHRNA5* rs16969968 and *CHRNA3* rs1051730 genotypes in 40 clinical trials with active arms (bupropion, varenicline, nicotine replacement therapy, or a combination of therapies) versus placebo. Although some evidence of pharmacological treatment by genotype interactions was found, most analyses failed to provide evidence of a differential response to treatment by genotype. The authors did not identify any widespread differential effects of smoking cessation pharmacotherapies based on genotype. The quality of evidence was moderate.

As highlighted here, treatment strategies for smoking cessation should be tailored to individual smokers based on their specific needs and biology (precision medicine). This study aimed to define proper strategies for starting treatment and combining drugs. Defining the best treatment strategy is recurrently discussed in the consensus of specialists and medical societies, such as the Consensus of the American College of Cardiology^10^ and the Guide of the American Thoracic Society^11^. There is a lack of data for consistently indicating drug combinations for smokers. The strategy of starting treatment with the most effective drug, varenicline, and adding bupropion at Week 4 when smoking cessation was not achieved, led to an excellent smoking cessation rate and reduced the chances of unnecessary drug administration and increased adverse events. Another important contribution of this study was the increased success rate after bupropion add-on at 150 mg/day instead of 300 mg/day, used in previous drug combination studies^31,32^. This treatment regimen led to 74% smoking cessation rates within 8 to 12 weeks.

Bear in mind that such rates agreed with those obtained in a previous observational study with smokers treated on an outpatient basis with the same pattern of drug prescription associated with the ‘cue restricted smoking’ behavior technique^25^ (77%). To the best of our knowledge, smoking cessation rates as high as those achieved in previous studies within 12 weeks of treatment^10,11,25^ have not been achieved elsewhere.

The role of a prediction variable like the delta of consumption is to guide treatment and allow the identification of smokers who would benefit from combination therapy at the intermediate phase of their treatment, increasing its efficacy. The possibility of testing this variable in future studies in smoking cessation is key to increasing abstinence rates all over the world. To do this, smoking cessation studies must evaluate outcome rates during treatment, especially at Week 4, like in this trial and the one by Scholz et al.^25^.

It is necessary to consider the importance of using effective drugs for smoking cessation. It is also important to recognize how behavioral techniques can increase abstinence rates, mainly because the number of smoking cessation drugs currently available is very limited. In addition, nowadays, there are no genomic markers useful in predicting better responses to smoking cessation treatment using pharmacotherapy.

### Limitations

This study is limited by its single-center and partially blind design (participants and medical staff were blind to varenicline administration). Subgroup analyses showed statistically significant and clinically relevant differences in several aspects, although without the power of sample calculation designed for the primary endpoint. Also, it is worth mentioning that the population under study was predominantly White and of Caucasian origin. Despite these limitations, we hope our results can be further supported by additional studies applying our methods for smoking cessation treatment.

## CONCLUSIONS

Although the genetic markers did not contribute to smoking cessation treatment, we showed that starting smoking cessation treatment with the most effective drug, varenicline, and then adding bupropion at Week 4 for those who were not able to achieve smoking cessation by then, led to an excellent smoking cessation rate and reduced the chances of unnecessary drug administration and increased adverse events.

## Supplementary Material



## Data Availability

The data supporting this research are available from the authors on reasonable request.
