# Supplementary material for: Efficacy of bupropion and varenicline genetic markers in choosing pharmacological treatment for smoking cessation, and implications for combining drugs: A randomized controlled trial – GENTSMOKING
Source: Tob Induc Dis. 2024 Apr 16;22:10.18332/tid/186072. doi: 10.18332/tid/186072 (PMC11019925; doi:10.18332/tid/186072)
Supplement: Supplementary file 1 [file TID-22-62-s1.pdf]

Supplemental Table 1. Abstinence rates between different subgroups considering polymorphism favorable markers and drug used. Logistic regression model

	Abstinence in week 4		OR (IC95%)	p
	No	Yes		
**Comparisons between different subgroups**				
**1 – each genetic subgroups vs control group**				0.001
Genetic favorable bupropion – drug used bupropion	81 (81.0)	19 (19.0)	0.31 (0.17 a 0.56)	<0.001
Genetic favorable varenicline – drug used varenicline	36 (57.1)	27 (42.8)	1.00 (0.56 a 1.78)	0.991
Genetic not favorable for both- both drugs used	11 (52.4)	10 (47.6)	1.21 (0.49 a 3.00)	0.683
Control group – drug used varenicline	101 (57.1)	76 (42.9)	reference	-
**2 – favorable genetic marker to varenicline vs not favorable marker in all varenicline users**				
Polymorphism favorable to varenicline – drug used varenicline	111 (57.8)	81 (42.2)	0.86 (0.46 a 1.63)	0.648
Polymorphism not favorable to varenicline – drug used varenicline	26 (54.2)	22 (45.8)	reference	-

p - descriptive level of the logistic regression model; OR - odds ratio; 95% CI - 95% confidence interval

Supplemental Table 2. Comparison of the efficacy of different strategies using combinations of varenicline and bupropion drugs.

	Abstinence continues between week 8-12		p
	N (%)	IC95%	
Cohort group			
started varenicline + bupropion	15/21 (71.4)	47.8 to 88.7	
started varenicline and added bupropion	49/79 (62.2)	50.4 to 72.7	0.725
started bupropion and added varenicline	25/39 (54.1)	46.0 to 78.2	

*p - descriptive level of the logistic regression model; 95% CI - 95% confidence interval

Supplemental Table 3. Interruption of treatment according to the initial medication.

	Interruption of treatment		OR (IC95%)	p
	No	Yes		
**Drug**				<0,001
varenicline	215 (88.8)	25 (11.3)	Reference	-
bupropion	69 (69.0)	31 (31.0)	3.54 (1.98 a 6.35)	<0,001
varenicline+bupropion	17 (81.0)	4 (19.0)	1.86 (0.58 a 5.92)	0,296

p - descriptive level of the logistic regression model; OR- odds ratio; 95% CI - 95% confidence interval

Supplemental table 4 – Adverse events until week 4 according to drug used.

Participants n 361 Adverse Events	Medication until week 4		
	vareniclina 2 mg/day (n=240)	Bupropion 300 mg /day (n=100)	Both drugs (2mg/day +150mg/day) (n=21)
Interrupted the drug and discontinued treatment	2 (1%)	11 (11%)	1(5%)
Serious adverse event	0	0	0
Any adverse events	148 (61.6%)	61 (61%)	15 (71.4%)
Events reported			
nausea	104 (30%)	2(2%)	10 (47%)
constipation	10 (3%)	4 (4%)	0
dreams lived	13 (5%)	0	0
dry mouth	0	13(13%)	0
insomnia	11(4%)	27(27%)	5(24%)
sleepiness	3 (1%)	0	0
headache	2 (<1%)	5(5%)	0
irritability	0	2(2%)	0
Metallic taste	3 (1%)	1(1%)	0
no medication related	2 (<1%)	1(1%)	0
tremor	0	6(6%)	0

Supplemental Table 5- Adverse events after week 5 according to drugs used

Participants 301<br>Events	Adverse events from week 5 to week 12				
	varenicline 2mg/day (n=136)	bupropion 300 mg/dia (n=30)	Both drugs 2mg + 150 mg/day (n=17)	varenicline added bupropion 150mg. (n=79)	bupropion added varenicline 2mg. (n=39)
Discontinued treatment for adverse event	0	0	0	0	0
Interrupted Varenicline	1		0	1	0
Interrupted bupropion		1	0	6	1
Any adverse events	12(9%)	11 (36%)	3(17%)	23(29%)	20 (51%)
Events reported					
nausea	5 (4%)	0	2 (12%)	3(4%)	13 (33%)
constipation	4 (3%)	1(3%)	0	4(5%)	3(8%)
dizziness	0	0	0	1 (1%)	0
dreams lived	0	0	0	0	3(8%)
dry mouth	0	2(6%)	0	2 (2%)	0
insomnia	0	8 (27%)	1(6%)	9 (11%)	0
headache	1(<1%)	0	0	1(1%)	0
irritability	0	0	0	1(1%)	0
metallic taste	0	0	0	1(1%)	0
no medication related	0	1	0	0	0
flatulence	1(<1%)	0	0	1(1%)	1(2%)
allergy	0	0	0	1(1%)	0

© 2024 Gaya P.V. et al.
